# Bioactive Fused Pyrazoles Inspired by the Adaptability of 5-Aminopyrazole Derivatives: Recent Review

**DOI:** 10.3390/molecules30020366

**Published:** 2025-01-17

**Authors:** Dana M. Odeh, Mohanad M. Odeh, Taghrid S. Hafez, Ashraf S. Hassan

**Affiliations:** 1 Department of Pharmacy, Faculty of Pharmacy, Jadara University, P.O. Box 733, Irbid 21110, Jordan; 2 Department of Clinical Pharmacy and Pharmacy Practice, Faculty of Pharmaceutical Sciences, The Hashemite University, P.O. Box 330127, Zarqa 13133, Jordan; mohanad_odeh@hu.edu.jo; 3 Organometallic and Organometalloid Chemistry Department, National Research Centre, Dokki, Cairo 12622, Egypt; taghridshafez@yahoo.com

**Keywords:** bioactive compounds, 5-aminopyrazole, fused pyrazoles, schiff bases, drugs

## Abstract

Heterocyclic compounds, especially those containing the pyrazole moiety, are highly significant in organic chemistry and possess remarkable and diverse biological properties. The 5-aminopyrazole derivatives are key starting materials for the synthesis of numerous bioactive compounds such as pyrazolopyridine, pyrazolopyrimidine, pyrazoloquinazoline, and pyrazolotriazine derivatives. Many compounds inspired by the 5-aminopyrazole derivatives possess a wide spectrum of biological activities and medicinal applications such as antioxidants, anticancer agents, enzyme inhibitors, antimicrobials, and anti-tuberculosis activities. This review summarizes the recently reported synthesis methods and biological activities of fused pyrazole and pyrazole-based derivatives based on 5-aminopyrazole compounds within the last 5 years (2020 to present). One of the important goals of this review is to illustrate future strategies for the design, development, and utilization of pyrazole products as potent drugs.

## 1. Introduction

Free radicals damage cells and can cause diseases such as cancer. They also contribute to aging, which is associated with some diseases such as diabetes, Alzheimer’s, and cardiovascular diseases. Therefore, antioxidant agents are very important to ameliorate the harmful impact of oxidative stress. Various types of antioxidant agents can be classified in several ways. Based on size, antioxidants are classified into small or large molecules. Vitamin C, vitamin E, and glutathione (GSH) are examples of small-molecule antioxidants. On the other hand, enzymes (SOD, CAT, and GPx) and sacrificial proteins (albumin) are the main examples of large-molecule antioxidants [1,2].

Cancer is a genetic disease in which the body’s cells grow uncontrollably. There are many things that can cause cancer, such as errors that occur during cell division, DNA damage, and genetic inheritance from parents [3,4].

Diabetes is a 21st-century challenge and is marked by a defect in the pancreas function for insulin secretion [5]. The glucose level in the body is regulated by the hormone insulin [6]. Medical research indicates a relationship between diabetes and obesity [7].

Alzheimer’s disease (AD) is prevalent in Arab nations and is a neurodegenerative disorder that causes dementia [8,9]. Medical studies indicate a relationship between diabetes mellitus, cardiovascular disease, and an increased risk of Alzheimer’s disease and dementia [10,11,12].

The interaction between humans and diverse microorganisms, such as bacteria or fungi, can lead to infectious diseases. To treat these diseases, antimicrobial agents (antibiotic drugs) are used to inhibit the growth of the microbes or kill them [13]. In the last few decades, administered antimicrobial medicines have become less effective due to microbial transformations. Therefore, multidrug resistance in bacteria (MDRB) has become a global health problem [14].

Heterocyclic compounds are highly significant in organic chemistry and play a significant part in drug development programs [15]. They are particularly valued in medicinal chemistry for their diverse properties and applications [16]. Nitrogen-containing heterocycles are highly significant due to their various applications in life and material sciences [17]. Some derivatives of nitrogen-containing heterocycles possess various biological applications [18,19,20,21]. The pyrazole motif is a five-membered heterocyclic ring with two adjacent nitrogen atoms in the 1- and 2-positions [22]. Many compounds tethered to the pyrazole scaffold and fused pyrazole derivatives possess a wide spectrum of biological activities and medicinal applications such as anticancer, antiviral, anti-inflammatory, antioxidant, and antibacterial properties [23,24,25].

**Figure 1:**
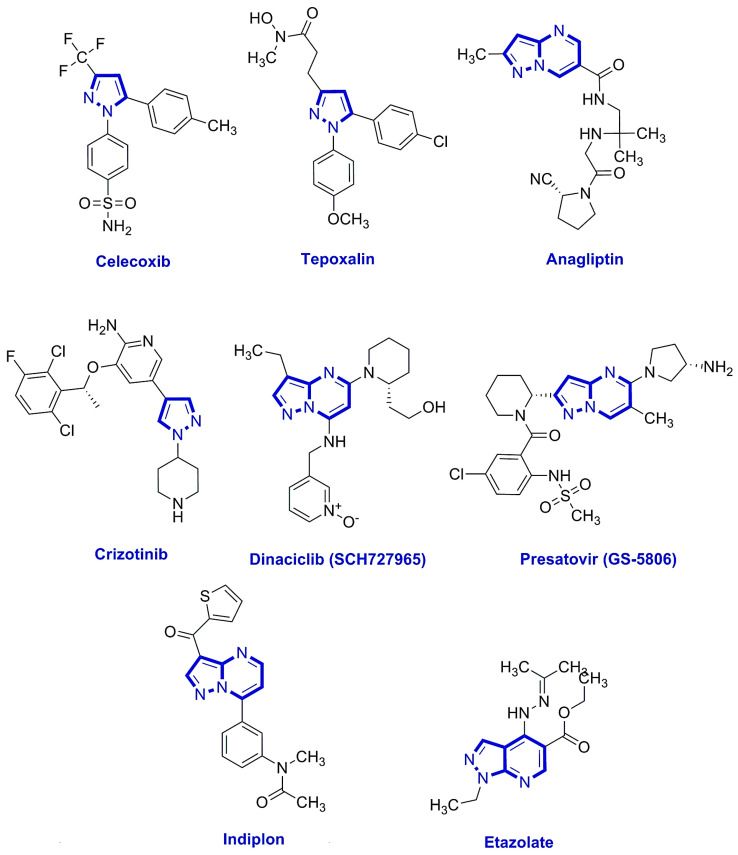
Structure of some examples of pyrazole drugs and fused pyrazole drugs.

Some examples of pyrazole drugs and fused pyrazole drugs that have been discovered or approved include celecoxib, tepoxalin, crizotinib, anagliptin, dinaciclib (SCH727965), indiplon, and presatovir (GS-5806) [26,27] (Figure 1), consequently confirming the pharmacological value of the pyrazole and fused pyrazole derivatives.

Celecoxib is an analgesic and a non-steroidal anti-inflammatory drug (NSAID) approved by the FDA in December 1998 and used for the treatment of osteoarthritis, rheumatoid arthritis, and acute pain [28]. Celecoxib is an inhibitor of the cyclooxygenase-2 (COX-2) enzyme, which reduces pain and inflammation. The COX-2 enzyme is heavily expressed in inflamed tissues [29]. By targeting the COX-2 enzyme, celecoxib reduces the synthesis of inflammatory mediators such as prostaglandin E2 (PGE2), thromboxane (TXA2), prostaglandin D2 (PGD2), prostacyclin (PGI2), and prostaglandin F2 (PGF2), leading to pain and inflammation relief [30].

Tepoxalin is a non-steroidal anti-inflammatory drug approved for veterinary medicine by the FDA in 1998 [31]. Tepoxalin is used for pain and inflammation relief through the inhibition of both cyclooxygenase-2 (COX-2) and 5-lipoxygenase (5-LOX) enzymes [32].

Anagliptin is an anti-diabetic medication that has been licensed in Japan since 2012. It is used for type 2 diabetes mellitus treatment through the inhibition of dipeptidyl peptidase-4 enzyme (DPP-4 enzyme) [33].

Crizotinib is an anti-cancer medication approved by the FDA in January 2021 for non-small cell lung carcinoma (NSCLC) treatment through the inhibition of both anaplastic lymphoma kinase (ALK) and c-ros oncogene 1 (ROS1) [34,35].

Dinaciclib (SCH727965) is an anti-cancer medication and a second-generation multi-CDK inhibitor. Dinaciclib, in combination with aprepitant, is used for the treatment of advanced malignancies by inhibiting cyclin-dependent kinases (CDK1, CDK2, CDK5, and CDK9) and anti-apoptotic BCL-XL and BCL2 proteins [36,37,38].

Presatovir (GS-5806) is an antiviral candidate used for the therapy of respiratory syncytial virus (RSV) by blocking the virus’ cell fusion process. In Phase II clinical trials, presatovir has shown promising results [39].

Indiplon is a sedative-hypnotic. It functions by improving the inhibitory neurotransmitter GABA’s action through binding to the α1 subunits of GABA-A receptors in the brain [40].

Etazolate is a selective, positive GABA-A receptor modulator that has completed phase II clinical trials in patients with Alzheimer’s disease. Also, Etazolate is a selective phosphodiesterase-4 inhibitor. Moreover, Etazolate showed anxiolytic and antidepressant activities [41,42].

Based on the above facts, as well as our continued evaluation of nitrogenous derivatives [43,44,45,46,47,48,49,50,51,52,53,54,55], the main objective of this review is to summarize the recently reported synthesis of fused pyrazole derivatives and compounds tethered to the pyrazole scaffold based on 5-aminopyrazole derivatives with various biological activities within the last 5 years (2020 till present). This study focuses on classifying the compounds into fused pyrazole derivatives and pyrazole-based compounds to demonstrate their various potential biological activities.

## 2. 5-Aminopyrazole Synthetic Strategies

There are various synthetic strategies for the preparation of 5-aminopyrazole derivatives. In this section, we will document and present some general examples of the synthetic routes to 5-aminopyrazole derivatives.

**Scheme 1:**
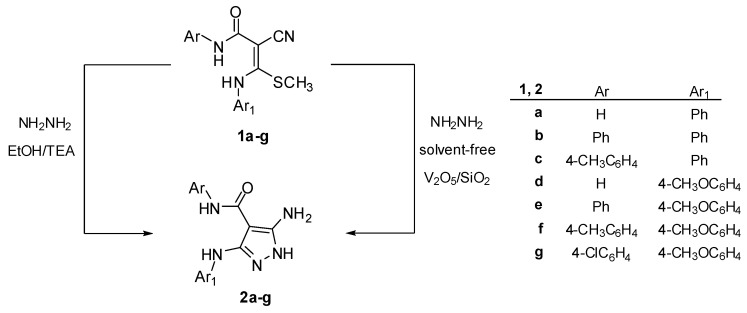
The synthesis route of 5-aminopyrazole derivatives **2a**–**g**.

Hassan et al. reported the synthesis of 5-aminopyrazoles **2a**–**g** (75–85% yields) through the reaction of ketene *N*, *S*-acetals **1a**–**g** with hydrazine hydrate in EtOH/TEA (Scheme 1) [56]. Furthermore, Khatab et al. synthesized the same 5-aminopyrazole derivatives **2a**–**g** (88–95% yields) via catalytic reaction of **1a**–**g** with hydrazine hydrate under solvent-free conditions using V_2_O_5_/SiO_2_ as a heterogeneous catalyst (Scheme 1) [57].

**Scheme 2:**
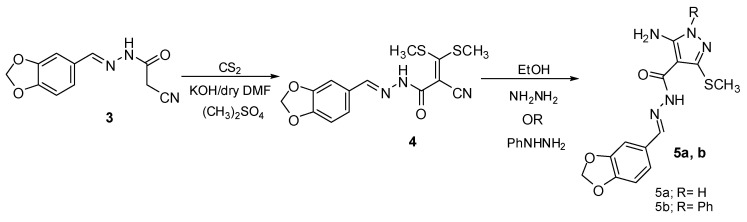
The synthesis route of 3-methylthio-5-aminopyrazoles **5a**,**b**.

In 2023, Hassan and Al-Sabawi reported the synthesis of 3-methylthio-5-aminopyrazoles **5a** and **5b** (yielding 45% and 52%, respectively) through two steps: the first step involves the reaction of cyanoacetic acid benzo[1,3]dioxol-5-ylmethylene hydrazide (**3**) with CS_2_ in KOH/dry DMF, followed by treatment with dimethyl sulfate (CH_3_)_2_SO_4_ to form *bis*(methylthio)acrylohydrazide derivative **4**. The second step involves the reaction of derivative **4** with hydrazine hydrate or phenylhydrazine in EtOH/TEA (Scheme 2) [58].

**Scheme 3:**
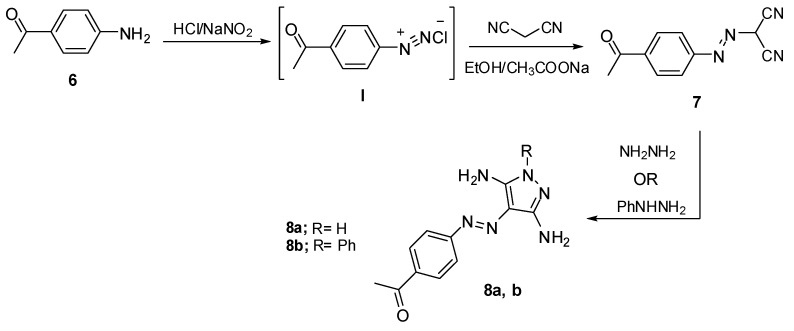
The synthesis route of 3, 5-diaminopyrazole derivatives **8a**,**b**.

2-[(4-Acetylphenyl)diazenyl]malononitrile (**7**) was produced via the treatment of *p*-aminoacetophenone (**6**) with HCl/NaNO_2_ and then with malononitrile in EtOH/CH_3_COONa solution. This compound, 2-[(4-acetylphenyl)diazenyl]malononitrile (**7**), was then treated with hydrazine hydrate in ethanol or phenylhydrazine in glacial acetic acid to produce 3, 5-diaminopyrazole derivatives **8a** and **8b** (yielding 42% and 64%, respectively) (Scheme 3) [59].

**Scheme 4:**
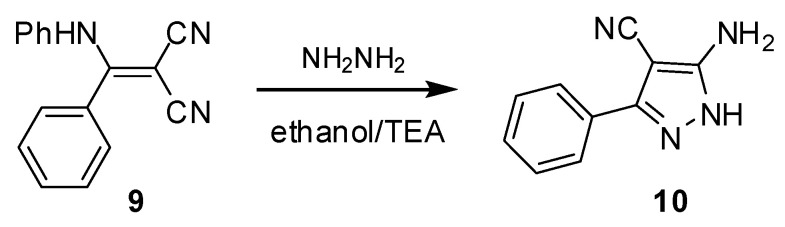
The synthesis route of 5-aminopyrazole-4-carbonitrile derivative **10**.

Treatment of 2-(phenyl(phenylamino)methylene)malononitrile (**9**) with hydrazine hydrate in ethanol/TEA resulted in ring closure, producing 5-aminopyrazole-4-carbonitrile derivative **10** (84% yield) (Scheme 4) [60].

**Scheme 5:**
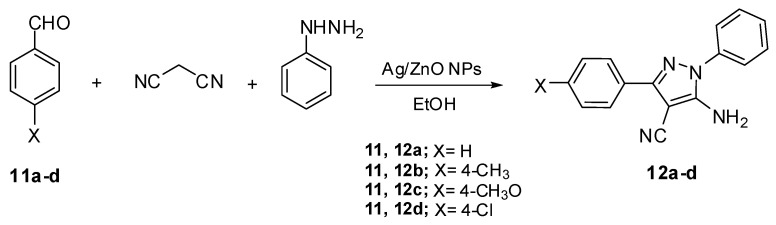
The synthesis route of 5-amino-3-aryl-1*H*-pyrazole-4-carbonitrile derivatives **12a**–**d**.

Yadav and his co-workers synthesized the 5-amino-1*H*-pyrazole-4-carbonitrile derivatives **12a**–**d** (89–94% yields) via catalytic reaction of aldehydes **11a**–**d**, malononitrile, with phenylhydrazine in ethanol using Ag/ZnO NPs (Scheme 5) [61].

**Scheme 6:**
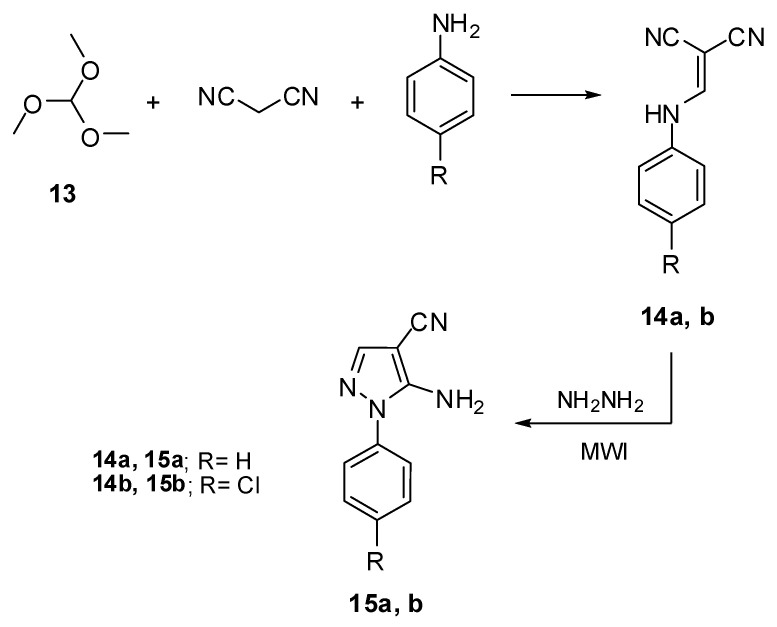
The synthesis route of 5-aminopyrazole-4-carbonitrile derivatives **15a**,**b**.

The condensation of the three components malononitrile, trimethoxymethane (**13**), and aromatic amines produced 3-aryl-2-cyanoacrylonitrile **14a** and **14b**. These compounds were then subjected to treatment with hydrazine hydrate under microwave irradiation (MWI) to yield two derivatives of 5-aminopyrazole-4-carbonitrile **15a** and **15b** (Scheme 6) [62].

**Scheme 7:**
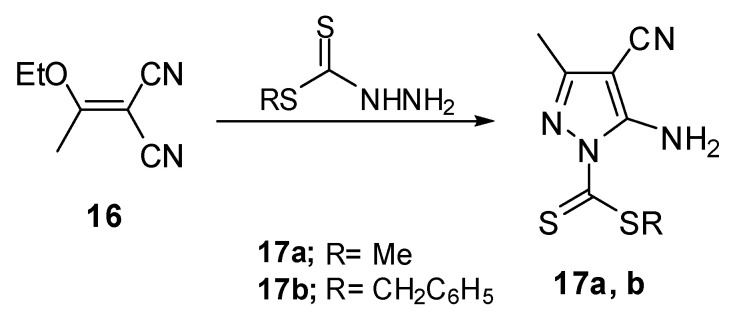
The synthesis route of 5-aminopyrazole-4-carbonitrle derivatives **17a**,**b**.

Nassar et al. reported the synthesis of 5-aminopyrazole-4-carbonitrile derivatives **17a**,**b** through the treatment of 2-(1-ethoxyethylidene)malononitrile (**16**) with methyl or benzyl hydrazinecarbodithioate in boiling ethanol (Scheme 7) [63].

## 3. The Reactivity of 5-Aminopyrazoles

**Figure 2:**
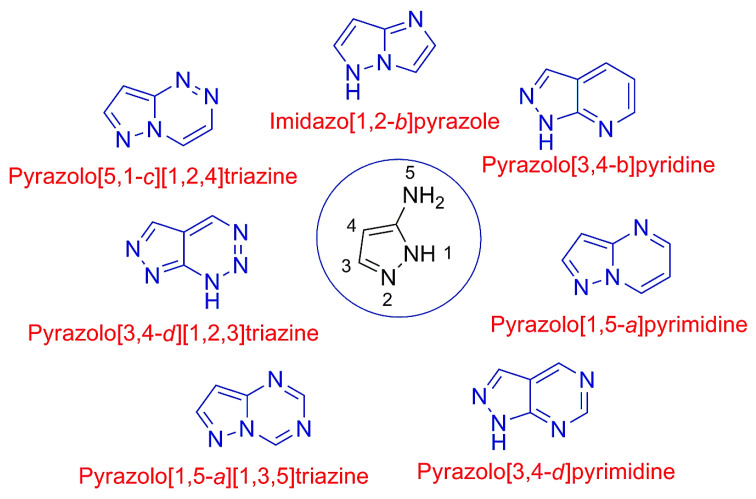
General structure of fused pyrazole inspired by 5-aminopyrazole derivative.

5-Aminopyrazole derivatives are key starting materials for the synthesis of numerous bioactive compounds such as pyrazolopyridines [64], pyrazolopyrimidines [65], pyrazoloquinazolines [66], imidazopyrazole [67], and pyrazolotriazines [68,69] (Figure 2).

The 5-aminopyrazole derivative is a polyfunctional compound that possesses three nucleophilic sites: 4-CH, 1-NH, and 5-NH2. These sites are utilized for the production of pyrazole compounds and fused pyrazole derivatives by cycloaddition and cyclization reactions with electrophiles.

## 4. Fused Pyrazoles: Synthesis and Biological Activities

### 4.1. Imidazo[1,2-b]pyrazole Derivatives

Imidazo[1,2-*b*]pyrazole derivatives possess a variety of therapeutic activities such as antimicrobial [70], anticancer [71,72] (e.g., against melanoma skin cancer (MSC) [73]), and anti-inflammatory [74]. Here are some examples of the synthesis and biological activities of imidazo[1,2-*b*]pyrazole derivatives.

**Scheme 8:**
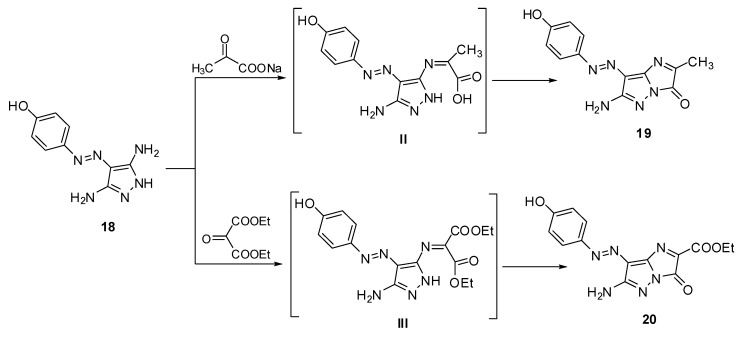
The synthesis route of imidazopyrazole derivatives **19** and **20**.

Imidazo[1,2-*b*]pyrazol-3-one derivatives **19** and **20** were synthesized as cyclooxygenase-2 (COX-2) inhibitors (anti-inflammatory agents). The imidazo[1,2-*b*]pyrazol-3-one derivatives **19** (75% yield) and **20** (86% yield) were prepared via the cyclization reaction of 3, 5-diamino pyrazole derivative **18** with sodium pyruvate and diethyl ketomalonate, respectively (Scheme 8). In vitro cyclooxygenase-2 enzymatic assay demonstrated that the two imidazo[1,2-*b*]pyrazol-3-one derivatives, **19** (IC_50_ = 295.39 ± 4.04 μM) and **20** (IC_50_ = 79.32 ± 1.32 μM), possess the least COX-2 suppressor potential compared with celecoxib (IC_50_ = 3.60 ± 0.07 μM) and meloxicam (IC_50_ = 7.58 ± 0.13 μM) [75].

**Scheme 9:**
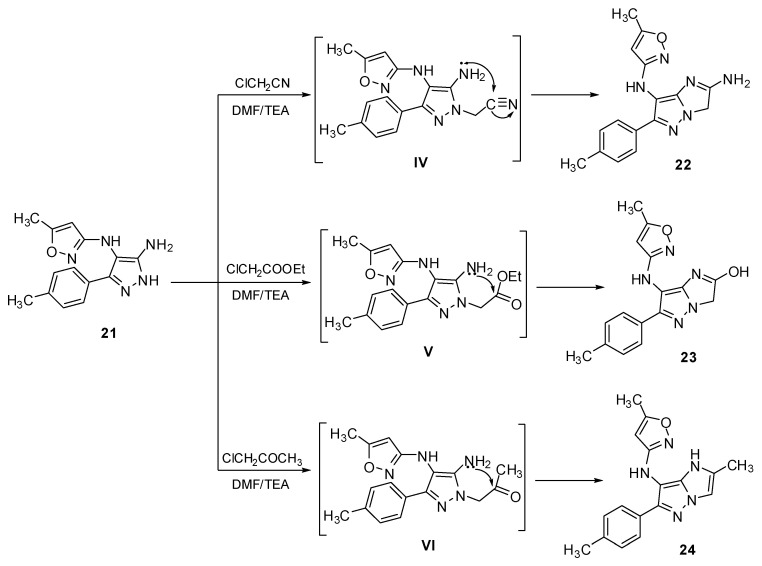
The synthesis route of imidazo[1,2-*b*]pyrazoles **22**–**24**.

In 2023, Alamshany and his coworkers prepared three derivatives of imidazo[1,2-*b*]pyrazole bearing an isoxazole moiety **22**–**24** (60–71% yields). These derivatives were produced through the cyclization reaction of 5-aminopyrazole derivative **21** with chloroacetonitrile, ethyl chloroacetate, and chloroacetone in a DMF/TEA solution, respectively (Scheme 9). The antioxidant activity results indicated that the two derivatives of imidazo[1,2-*b*]pyrazole, **22** and **23**, possess antioxidant biological properties with an inhibition percentage of 75.3% and 72.9%, respectively, compared to ascorbic acid with 89%. But, imidazo[1,2-*b*]pyrazole derivative **24** possesses low antioxidant activity (47.9%). On the other hand, the antimicrobial activity results indicated that derivative **22** (MIC: 0.03 µg/mL) was 65-fold more potent than ampicillin (MIC: 1.95 µg/mL) against *Escherichia coli* Gram −ve bacterium. It was also two-fold more potent than ampicillin (MIC: 0.98 µg/mL) against *Pseudomonas aeruginosa* Gram −ve bacterium (MIC: 0.49 µg/mL). Derivative **23** was equipotent to ampicillin against *Bacillus subtilis* Gram +ve bacterium and *Pseudomonas aeruginosa* Gram −ve bacterium (MIC values: 0.03 and 0.98 µg/mL, respectively). Finally, derivative **24** was inactive against bacteria and fungi [76].

**Scheme 10:**
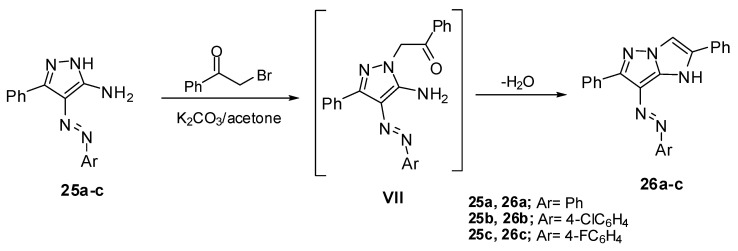
The synthesis route of aryl azo imidazopyrazole derivatives **26a**–**c**.

Aryl azo imidazo[1,2-*b*]pyrazole derivatives **26a**–**c** (yield about 72%) were synthesized via the cyclization reaction of 5-amino-3-phenyl-1*H*-pyrazole derivatives **25a**–**c** with 2-bromo acetophenone in refluxed K_2_CO_3_/acetone solution (Scheme 10). In vitro anticancer activity results indicated that the three aryl azo imidazo[1,2-*b*]pyrazole derivatives **26a**–**c** possess anticancer properties (IC_50_ = 6.1 ± 0.4 µM, 8.0 ± 0.5 µM, and 7.4 ± 0.3 µM, respectively) more potent than doxorubicin (IC_50_ = 10.3 ± 0.8 µM) against MCF-7 cancer cells [77].

**Scheme 11:**
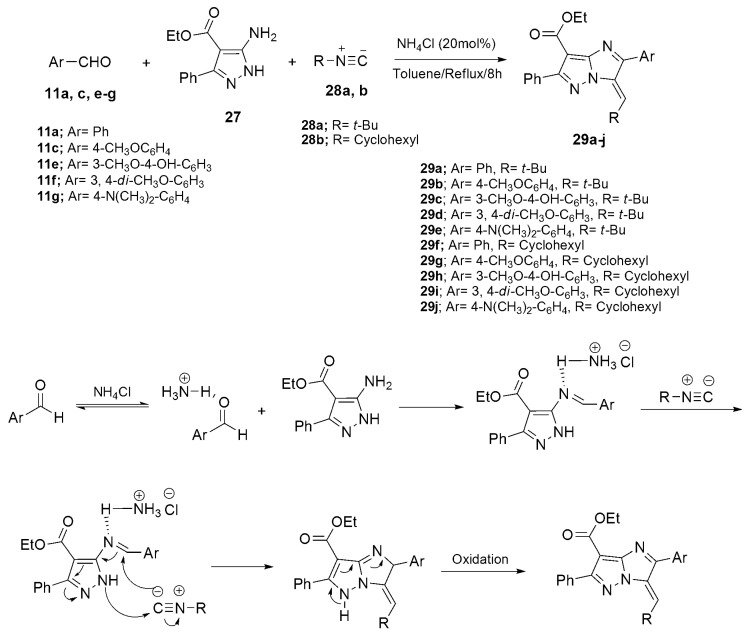
The synthesis route and the plausible reaction mechanism of imidazo[1,2-*b*]pyrazole-7-carboxylate derivatives **29a**–**j**.

Imidazo[1,2-*b*]pyrazole-7-carboxylate derivatives **29a**–**j** were designed and synthesized as candidates for the treatment of diabetes (anti-diabetic agents) through the inhibition of the α-glucosidase enzyme by Peytam and his coworkers. The target products were obtained via a one-pot three-component reaction. These derivatives **29a**–**j** (79–94% yields) were produced through the reaction of various aldehydes **11a**,**c**,**e**–**g** and 5-amino-1*H*-pyrazole-4-carboxylate derivative **27** with isocyanides **28a**,**b** in a toluene/NH_4_Cl (20 mol%) solution (Scheme 11). In vitro α-glucosidase inhibitory activity results demonstrated that derivative **29h** (IC_50_ = 95.0 ± 0.5 µM) was 8-fold more potent than acarbose (IC_50_ = 750 ± 1.5 µM) [78].

### 4.2. Pyrazolo[3,4-b]pyridine Derivatives

A large number of pyrazolo[3,4-*b*]pyridine derivatives have been reported, exhibiting various activities such as antioxidant [79], anticancer [80,81], PIM-1 kinase inhibitors in breast cancer MCF-7 cells [82], potent p53-MDM2 inhibitor [83,84], CDK2 inhibitor [84], antimicrobial [85], anti-tuberculosis [86], and DHFR inhibitor [87]. Herein, we will demonstrate some examples of the synthesis and biological activities of pyrazolo[3,4-*b*]pyridine derivatives.

**Scheme 12:**
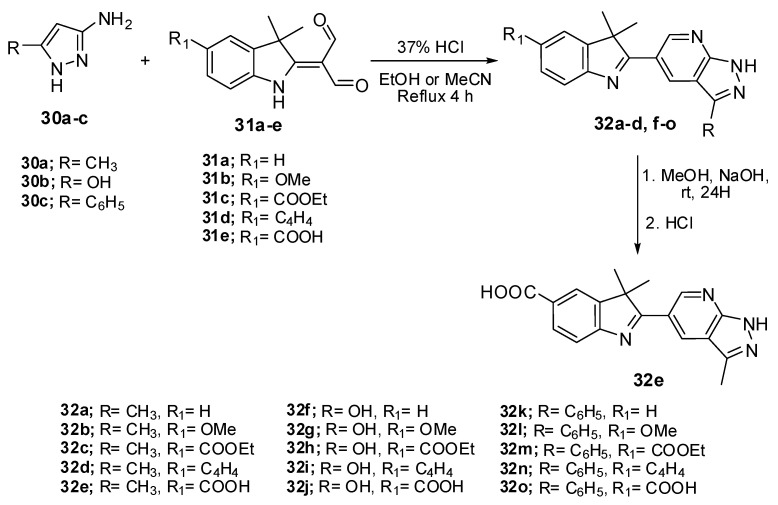
The synthesis route of indoleninyl-pyrazolo[3,4-*b*]pyridine derivatives **32a**–**o**.

Basir et al. (2024) reported the design and synthesis of a new series of indoleninyl pyrazolo[3,4-*b*]pyridine derivatives **32a**–**o** (22–96% yields), except for **32e**, decorated with indolenines via the acid-catalyzed cyclo-condensation of 5-aminopyrazoles **30a**–**c** with 1,3-dialdehyde indolenine derivatives **31a**–**e**. Derivative **32e** (83% yield) was obtained through the base hydrolysis of derivative **32c** (Scheme 12). The in vitro cytotoxic activity (IC_50_) of some indoleninyl pyrazolo[3,4-*b*]pyridine derivatives **32k**–**o** against HCT 116, HT-29, and CCD-18Co cells was assessed. The results demonstrated that derivative **32n** among the tested products possesses cytotoxic activity toward the HCT 116 and HT-29 cell lines relative to cisplatin as a positive standard [88].

**Scheme 13:**
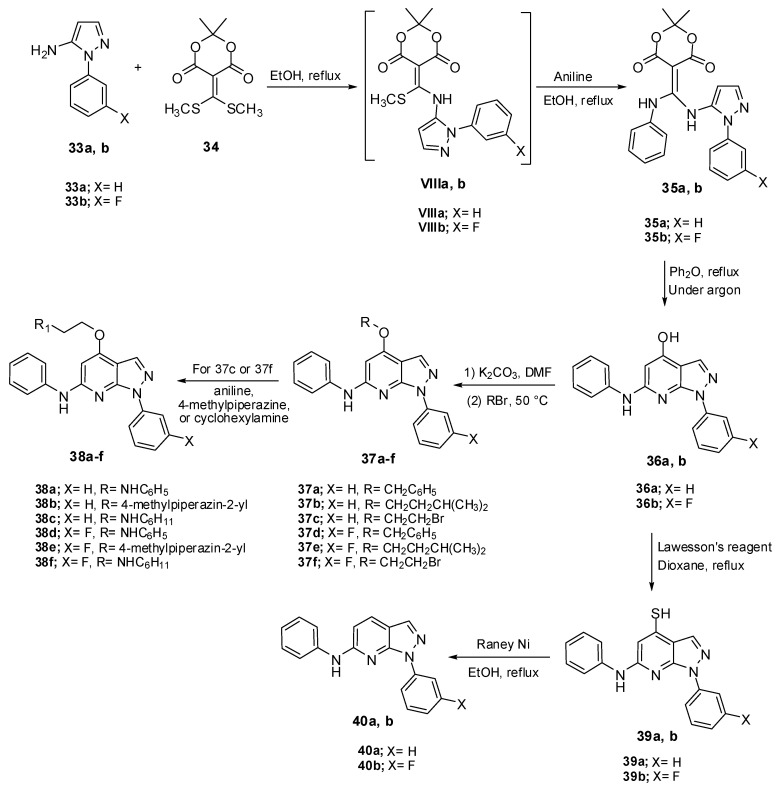
The synthesis route of five series of pyrazolo[3,4-*b*]pyridine derivatives **36a**,**b**, **37a**–**f**, **38a**–**f**, **39a**,**b**, and **40a**,**b**.

Five series of pyrazolo[3,4-*b*]pyridine derivatives **36a**,**b**, **37a**–**f**, **38a**–**f**, **39a**,**b**, and **40a**,**b** were prepared based on 5-aminopyrazoles **33a**,**b**. By treating **33a**,**b** with Meldrum’s acid dimethylthiomethylene derivative **34**, dioxanediones **VIIIa**,**b** were obtained. These intermediates then reacted with aniline to yield dioxanedione derivatives **35a**,**b**. Finally, derivatives **35a**,**b** underwent thermal cyclization and transformation into the pyrazolopyridinols **36a**,**b** (with 85% yield for each). The treatment of pyridinones **36a**,**b** with K_2_CO_3_/appropriate bromides formed the corresponding **37a**–**f** (65–98% yields). The two bromides **37c** and **37f** were reacted with aniline, 4-methylpiperazine, or cyclohexylamine to produce the amino derivatives **38a**–**f** (65–98% yields). The treatment of pyridinones **36a**,**b** with Lawesson’s reagent formed thiols **39a** and **39b** (yielding 20% and 18%, respectively). Finally, the reduction of thiols **39a**,**b** using Raney nickel as a catalyst yielded the corresponding pyrazolopyridines **40a** (20% yield) and **40b** (18% yield) (Scheme 13). Preliminary experiments were performed to determine the in vitro cytotoxic activity (IC_50_) of compounds **36a**,**b**, **37a**–**f**, **38a**–**f**, **39a**,**b**, and **40a**,**b**, toward the prostatic PC-3 and colon HCT116 human cancer cell lines. The results demonstrated that the four derivatives **38b**, **38c**, **38e**, and **38f** possess cytotoxic activity when compared to doxorubicin as a positive standard. Subsequently, the four active derivatives **38b**, **38c**, **38e**, and **38f** were evaluated for their activities against a breast cancer cell line (EO771), a pancreas cancer cell line (KPC), and one non-cancerous murine cell line of mouse embryonic fibroblasts (MEFs). The results demonstrated that the three derivatives **38b**, **38c**, and **38e** were found to be non-toxic. Therefore, the in vivo efficacy of **38b**, **38c**, and **38e** in a mouse breast cancer model was evaluated; the derivative **38b** displayed a powerful inhibition of tumor growth and induced apoptosis [89].

**Scheme 14:**
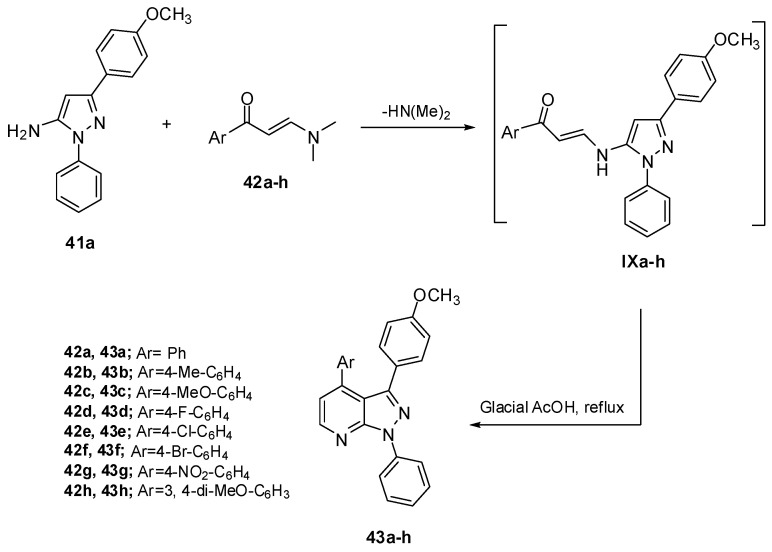
The synthesis route of pyrazolo[3,4-*b*]pyridine derivatives **43a**–**h**.

**Scheme 15:**
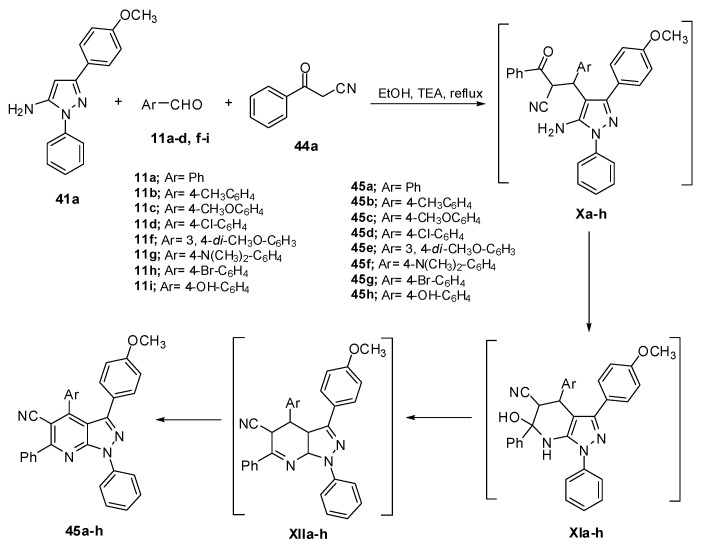
The synthesis route of pyrazolo[3,4-*b*]pyridine-5-carbonitrile derivatives **45a**–**h**.

Almansour et al. synthesized a series of pyrazolo[3,4-*b*]pyridine derivatives **43a**–**h** (66–76% yields) through the cyclo-condensation reaction of 5-aminopyrazole derivative **41a** with prop-2-en-1-one derivatives **42a**–**h** (Scheme 14). Additionally, the same team reported the synthesis of pyrazolo[3,4-*b*]pyridine-5-carbonitrile derivatives **45a**–**h** (63–83% yields) via a one-step three-component reaction of 3-oxo-3-phenylpropanenitrile (**44a**) with 5-aminopyrazole derivative **41a** and aromatic aldehydes **11a**–**d**,**f**–**i** (Scheme 15). The in vitro cytotoxic activity (IC_50_) of pyrazolo[3,4-*b*]pyridine derivatives **43a**–**h** and **45a**–**h** against HeLa, MCF-7, and HCT-116 cells was assessed. The results demonstrated that two derivatives **43a** and **45h** possess significant anticancer activity. Derivative **43a** (IC_50_ = 2.59 µM) possesses the highest activity among other derivatives against HeLa cells when compared with doxorubicin (IC_50_ = 2.35 µM). Derivative **45h** disclosed its cytotoxicity against MCF-7 and HCT-116 cells with IC_50_ values of 4.66 and 1.98 µM compared to doxorubicin with IC_50_ = 4.57 and 2.11 µM, respectively. The results of the cell cycle and apoptosis experiments demonstrated that product **43a** caused cell cycle arrest at the S phase in HeLa cells, while product **45h** induced cell cycle arrest at the G2/M phase in MCF-7 cells and at the S phase in HCT-116 cells. Further, product **43a** caused a significant level of early and late apoptosis in HeLa cells, while product **45h** caused apoptosis in MCF-7 and HCT-116 cells. Also, the two products **43a** and **45h** presented good safety profiles and inhibition activity towards CDK2 and CDK9 enzymes when compared with ribociclib, a positive control [90].

**Scheme 16:**
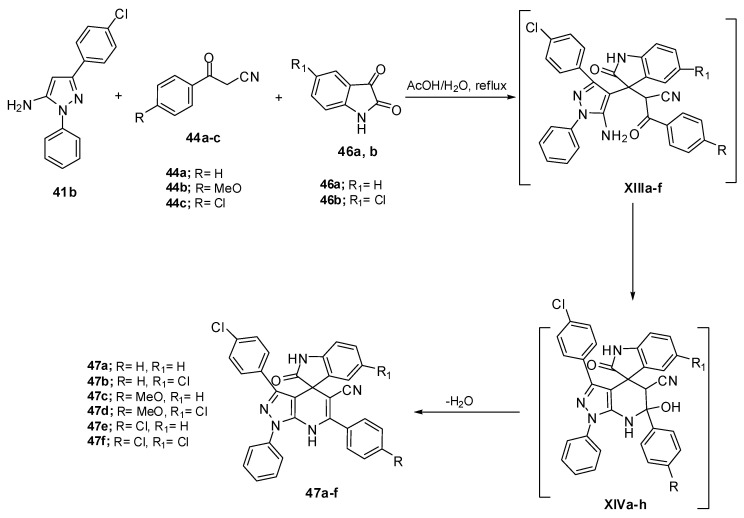
The synthesis route of spiro pyrazolopyridine derivatives **47a**–**f**.

Farahat et al. synthesized a novel series of spiro pyrazolo[3,4-*b*]pyridine derivatives **47a**–**f** (30–60% yields) via a one-pot three-component reaction of pyrazole derivative **41b** with benzoyl acetonitrile derivatives **44a**–**c** and indoline-2,3-diones **46a**,**b** (Scheme 16). The in vitro cytotoxic activity (IC_50_) of spiro pyrazolo[3,4-*b*]pyridine derivatives **47a**–**f** against HepG2 and HeLa cells was assessed. The results demonstrated that two derivatives **47a** and **47d** possess effective anticancer activity against HepG2 and HeLa cells with IC_50_ values of 4.2 and 5.9 µM, respectively, compared to doxorubicin with IC_50_= 1.7 and cisplatin with IC_50_ = 4.8 µM, respectively. The results of the cell cycle and apoptosis experiments demonstrated that product **47a** caused a reduction in the G1 phase with a significant arrest in the G2/M phase in HepG2 cells. Furthermore, product **47a** is a good apoptotic inducer and showed good inhibition of p38α kinase with an IC_50_ of 0.13 μM compared to SB 202190 as a positive control [91].

### 4.3. Pyrazolo[1,5-a]pyrimidine Derivatives

Pyrazolo[1,5-*a*]pyrimidines have been considerably investigated due to their biological and pharmacological significance as antioxidants, anti-Alzheimer’s agents [92], antimicrobial compounds [93,94,95], anticancer agents with potent CDK1 inhibitors [96], potent carbonic anhydrase I and II inhibitors [97], α-glucosidase inhibitors [98], anti-inflammatory agents [99,100], and immunomodulatory agents [101]. Here, we will showcase a selection of examples of the synthesis and biological activities of pyrazolo[1,5-*a*]pyrimidine derivatives.

**Scheme 17:**
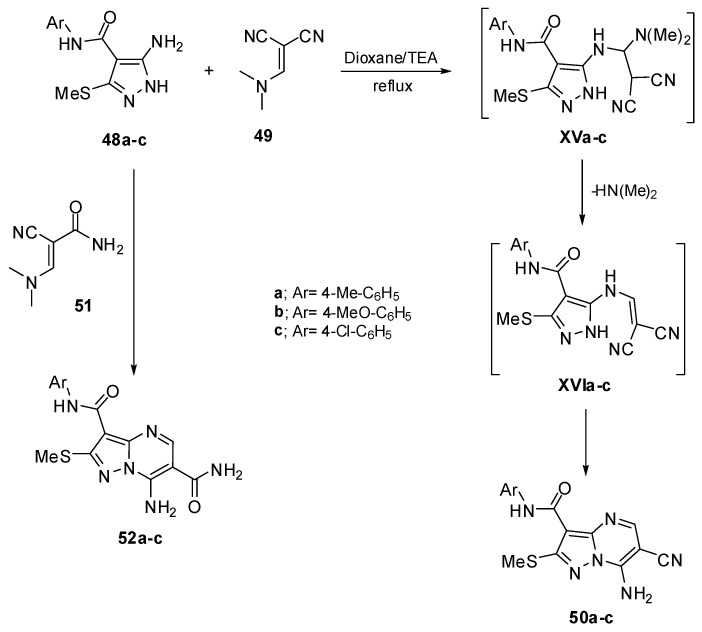
The synthesis route of 7-amino-pyrazolo[1,5-*a*]pyrimidine products **50a**–**c** and **52a**–**c**.

Hossan et al. prepared a new series of 7-amino-6-cyano-pyrazolo[1,5-*a*]pyrimidine products **50a**–**c** (73–81% yields) via the reaction of 5-amino-3-methylthio-pyrazole derivatives **48a**–**c** with 2-((dimethylamino)methylene)malononitrile (**49**). Furthermore, 7-amino-pyrazolo[1,5-*a*]pyrimidine-6-carboxamide products **52a**–**c** (56–64% yields) were prepared via the condensation reaction of 5-amino-3-methylthio-pyrazole derivatives **48a**–**c** with 2-cyano-3-(dimethylamino)acrylamide (**51**) (Scheme 17). The in vitro cytotoxic activity (IC_50_) of the two series of pyrazolo[1,5-*a*]pyrimidine derivatives **50a**–**c** and **52a**–**c** against HepG2, Hep-2, MCF-7, PC3, and normal WI38 cells were screened and the results were compared with 5-fluorouracil as a reference drug. The compounds possessed good cytotoxic effects on MCF-7 cells compared to the other cell lines [102].

**Scheme 18:**
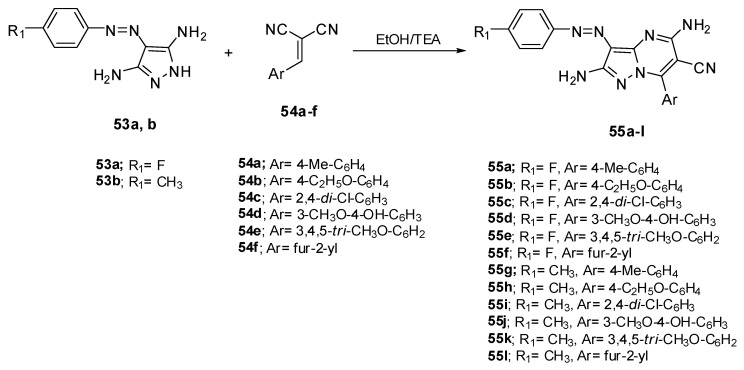
The synthesis route of 3-arylazo-pyrazolo[1,5-*a*]pyrimidine **55a**–**l**.

Abdulrahman et al. developed a new series of 3-arylazo-pyrazolo[1,5-*a*]pyrimidine **55a**–**l** (70–92% yields) as anticancer agents through the inhibition of PIM kinase. They achieved this by reacting 3, 5-diaminopyrazole derivatives **53a**,**b** with arylidenemalononitriles **54a**–**f** in an EtOH/TEA solution (Scheme 18). The in vitro cytotoxic activity (IC_50_) of pyrazolo[1,5-*a*]pyrimidine derivatives **55a**–**l** against HCT-116, HepG2, and MCF-7 cells was screened. Among the derivatives, the three compounds **55h**, **55j**, and **55l** possess noteworthy anticancer activities with IC_50_ values ranging from 1.26 to 3.22 μM. The results of the PIM kinase inhibition assessment (PIM-1, PIM-2, and PIM-3) demonstrated that the three derivatives are potent inhibitors of PIM-1 and PIM-2 but moderate inhibitors of PIM-3. The results of the cell cycle and apoptosis experiments demonstrated that product **55j** caused arrest at the G1 phase and induced apoptosis [103].

**Scheme 19:**
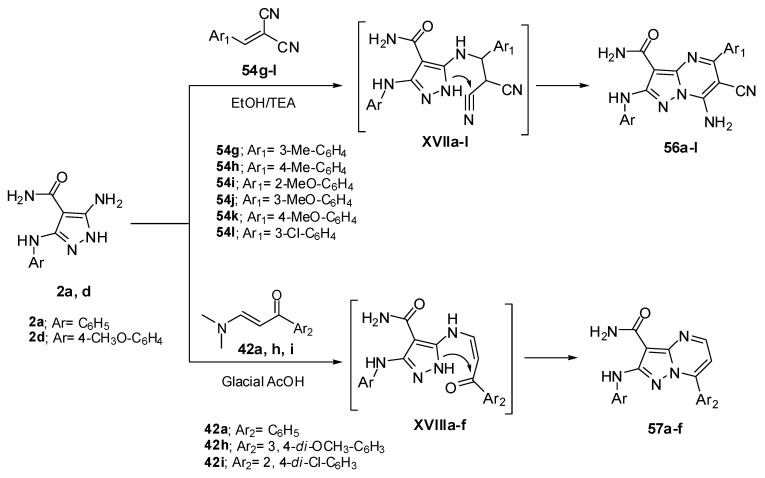
The synthesis route of pyrazolo[1,5-*a*]pyrimidine products **56a**–**l** and **57a**–**f**.

In 2023, Hassan et al. synthesized a series of 5-aryl-pyrazolo[1,5-*a*]pyrimidine products **56a**–**l** (73–80% yields) and 7-aryl-pyrazolo[1,5-*a*]pyrimidine products **57a**–**f** (63–79% yields) through the cyclo-condensation reaction of 5-amino-1*H*-pyrazoles **2a**,**d** with 2-(arylidene)malononitriles **54g**–**l** and 3-(dimethylamino)-1-aryl-prop-2-en-1-ones **42a**,**h**,**i**, respectively, (Scheme 19). The antioxidant, anti-diabetic, anti-Alzheimer’s, and anti-arthritic activities of 5-aryl-pyrazolo[1,5-*a*]pyrimidine products **56a**–**l** and 7-aryl-pyrazolo[1,5-*a*]pyrimidine products **57a**–**f** were evaluated. The evaluation results revealed that the product **56l** exhibited the highest antioxidant, scavenging, anti-diabetic, and anti-Alzheimer activities. The product **56i** presented the highest anti-arthritic activity. Also, the four products (**56c**, **56d**, **56g**, and **56h**) possess the same antioxidant activity [104].

**Scheme 20:**
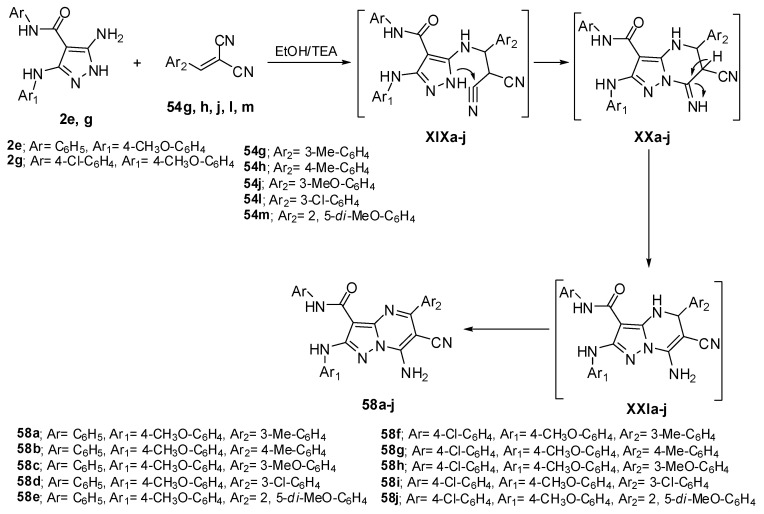
The synthesis route of pyrazolo[1,5-*a*]pyrimidine products **58a**–**j**.

In 2022, Hassan et al. reported the synthesis of new pyrazolo[1,5-*a*]pyrimidine products **58a**–**j** (71–80% yields) as antimicrobial agents. They achieved this by reacting two derivatives of 5-amino pyrazole **2e**,**g** with 2-(arylidene)malononitrile derivatives **54g**,**h**,**j**,**l**,**m** (Scheme 20). The in vitro antimicrobial activities against *Bacillus subtilis*, *Escherichia coli*, *Aspergillus niger*, and *Candida albicans* for the pyrazolo[1,5-*a*]pyrimidine products **58a**–**j** were screened. The antimicrobial evaluation results revealed that the three pyrazolo[1,5-*a*]pyrimidine products (**58a**, **58e**, and **58j**) possess a broad-spectrum antimicrobial activity when compared to the reference drugs (tetracycline, novobiocine, clotrimazole, and cyclohexamide) [105].

**Scheme 21:**
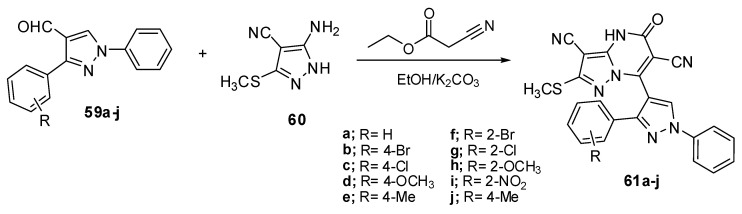
The synthesis route of pyrazolo[1,5-*a*]pyrimidine products **61a**–**j**.

A new series of dihydropyrazolo[1,5-*a*]pyrimidine products **61a**–**j** (74–81% yields) were synthesized as antimicrobial agents. The products were prepared by reacting 1*H*-pyrazole-4-carbaldehyde derivatives **59a**–**j** with 5-amino-1*H*-pyrazole-4-carbonitrile derivative **60** and ethyl cyanoacetate in an EtOH/K_2_CO_3_ solution (Scheme 21). All the products, **61a**–**j**, were tested for antimicrobial activity. The antimicrobial evaluation results revealed that the two products **61b** and **61i** possess powerful antimicrobial activities against *E. coli*, *S. Typhi*, *B. megaterium*, and *Micrococcus* spp. when compared to the reference drugs (streptomycin, ciprofloxacin, and nystatin) [106].

### 4.4. Pyrazolo[3,4-d]pyrimidine Derivatives

There is significant interest in synthesizing pyrazolo[3,4-*d*]pyrimidine derivatives due to their promising biological activities, including anti-cancer properties [107,108,109,110], potent c-Src kinase inhibitors [111], EGFR and VEGFR inhibitors [112], potential DHFR inhibitors [113], selective phosphodiesterase-5 (PDE5) inhibitors [114], antimicrobial effects [115,116], as well as antitubercular and anti-inflammatory activity [117]. Herein, we will demonstrate various models for the synthesis and biological applications of pyrazolo[3,4-*d*]pyrimidine derivatives.

**Scheme 22:**
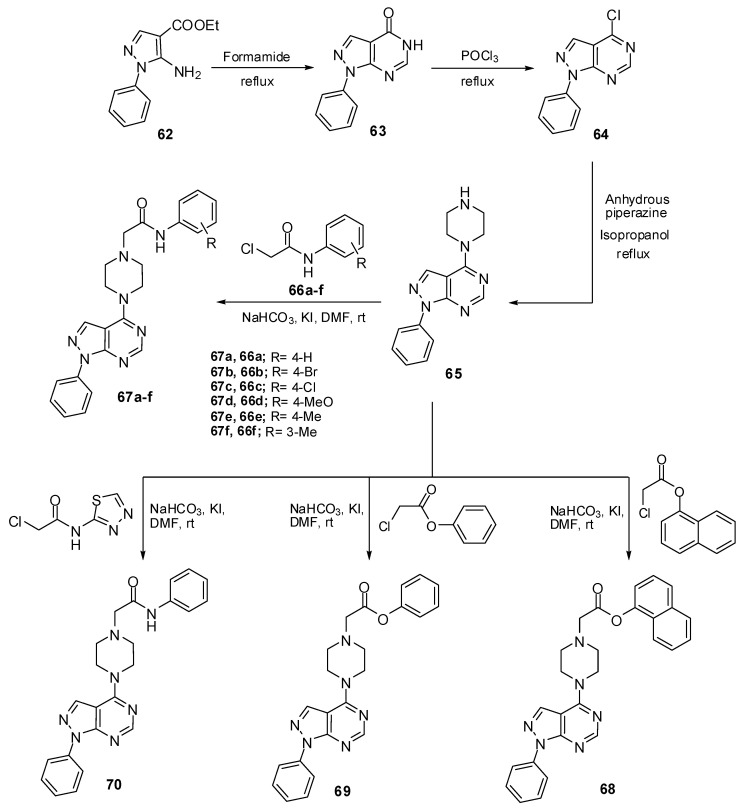
The synthesis route of 4-piperazine pyrazolo[3,4-*d*]pyrimidine derivatives **67a**–**f**, **68**–**70**.

The 4-piperazine pyrazolo[3,4-*d*]pyrimidine derivative **65** was synthesized in three steps. The first step involved the cyclization of ethyl 5-aminopyrazolo-4-carboxylate derivative **62** with formamide to yield 1-phenyl-pyrazolo[3,4-*d*]pyrimidin-4-one derivative **63** (70% yield). In the second step, product **63** was chlorinated using POCl_3_ to form 4-chloro-pyrazolo[3,4-*d*]pyrimidine derivative **64** (65% yield). Finally, in the third step, a reaction of product **64** with an excess of anhydrous piperazine was performed to produce 4-piperazine pyrazolo[3,4-*d*]pyrimidine derivative **65** (70% yield). The derivative **65** was utilized in the synthesis of *N*-aryl-2-(pyrazolo[3,4-*d*]pyrimidin-4-yl)piperazin-1-yl-acetamide derivatives **67a**–**f** (70–82% yields), naphthalen-1-yl 2-(pyrazolo[3,4-*d*]pyrimidin-4-yl)piperazin-1-yl-acetate derivative **68** (55% yield), and phenyl 2-(pyrazolo[3,4-*d*]pyrimidin-4-yl)piperazin-1-yl-acetate derivative **69** (50% yield) by reacting with 2-chloro-*N*-substituted phenyl acetamide derivatives **66a**–**f**, naphthyl chloroacetate, or phenyl chloroacetate, respectively. Also, 2-(pyrazolo[3,4-*d*]pyrimidin-4-yl)piperazin-1-yl-*N*-(1,3,4-thiadiazol-2-yl)acetamide derivative **70** (75% yield) was prepared by reacting 4-piperazine pyrazolo[3,4-*d*]pyrimidine derivative **65** with 2-chloro-*N*-(1,3,4-thiadiazol-2-yl)acetamide (Scheme 22). The anticancer evaluation results revealed that product **70** was tested in National Cancer Institute (NCI) full panel 5 dose assays and showed strong anticancer activity against most cell lines across nine subpanels. Products **67a**–**f** also displayed significant anticancer activity, especially against melanoma, leukemia, renal cancer, and breast cancer [118].

**Scheme 23:**
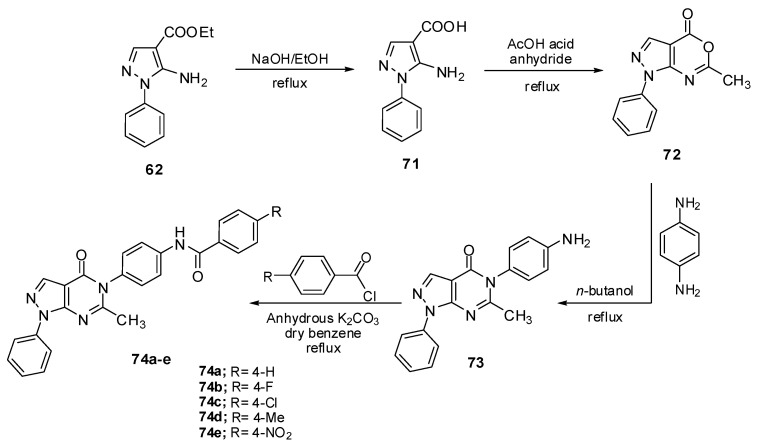
The synthesis route of pyrazolo[3,4-*d*]pyrimidine derivatives **74a**–**e**.

In 2023, Abdelhamed et al. reported the synthesis of a novel series of pyrazolo[3,4-*d*]pyrimidine derivatives. The ethyl 5-aminopyrazole-4-carboxylate derivative **62** underwent basic hydrolysis to produce the 5-aminopyrazole-4-carboxylic acid derivative **71**. Subsequently, product **71** was refluxed in CH_3_COOH acid anhydride to obtain the pyrazolo[3,4-*d*][1,3]oxazin-4(1*H*)-one derivative **72**. Finally, product **72** was reacted with *p*-phenylenediamine to yield the 5-(4-aminophenyl)-1*H*-pyrazolo[3,4-*d*]pyrimidine-4-one derivative **73** (45% yield). The target products, pyrazolo[3,4-*d*]pyrimidine benzamide derivatives **74a**–**e** (46–90% yields), were prepared by the reaction of 5-(4-aminophenyl)-1*H*-pyrazolo[3,4-*d*]pyrimidine-4-one derivative **73** with substituted benzoyl chloride (Scheme 23). The anticancer evaluation results revealed that products **74a**–**d** possess anticancer activity against breast cells, especially two types, MDA-MB-468 and T-47D. Remarkably, product **74b** demonstrated IC_50_ values of 3.343 ± 0.13 μM and 4.792 ± 0.21 μM against MDA-MB-468 and T-47D cells, respectively, compared to staurosporine as a reference drug. Additionally, it demonstrated potent inhibition of VEGFR-2 with an IC_50_ value of 0.063 ± 0.003 μM compared to sunitinib [119].

**Scheme 24:**
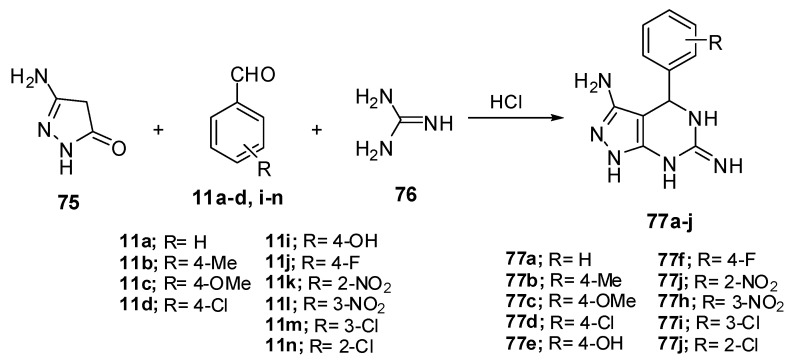
The synthesis route of 3-aminopyrazolo[3,4-*d*]pyrimidine derivatives **77a**–**j**.

In 2022, Dubal et al. reported the synthesis of 3-aminopyrazolo[3,4-*d*]pyrimidine derivatives **77a**–**j** (46–82% yields) through Biginelli condensation. They utilized 5-aminopyrazol-3-one derivative **75** and aromatic aldehydes **11a**–**c**,**i**–**n** with guanidine (**76**) in the reaction (Scheme 24). All the products, **77a**–**j**, were tested for antimicrobial activity. The antimicrobial evaluation results revealed that the products **77a**, **77c**, **77e**, **77i**, and **77j** possess powerful antimicrobial activities against *Streptococcus pyogenes*, *Staphylococcus aureus*, *Pseudomonas aeruginosa*, *Escherichia coli*, *Aspergillus niger*, and *Aspergillus clavatus* compared to the standard drugs used (amoxycillin, chloramphenicol, ciprofloxacin, norfloxacin, griseofulvin, and nystatin) [120].

### 4.5. Pyrazolo[1,5-a][1,3,5]triazine Derivatives

Pyrazolo[1,5-*a*][1,3,5]triazine derivatives exhibit anti-cancer [121], anticonvulsant [122], and antibacterial [123] activities. In this part, we will explore some synthesis methods and biological activities of pyrazolo[1,5-*a*][1,3,5]triazine derivatives.

**Scheme 25:**
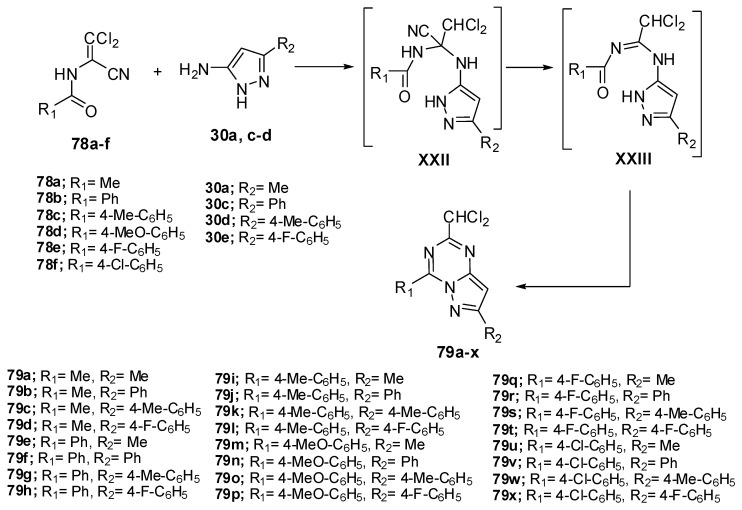
The synthesis route of 2-(dichloromethyl)pyrazolo[1,5-*a*][1,3,5]triazine derivatives **79a**–**x**.

The synthesis of 2-(dichloromethyl)pyrazolo[1,5-*a*][1,3,5]triazine derivatives **79a**–**x** (50–79% yields) was achieved through the heterocyclization reaction of *N*-(2,2-dichloro-1-cyanoethenyl)carboxamide derivatives **78a**–**f** with 5-aminopyrazole derivatives **30a**,**c**–**e** (Scheme 25). All the products, 2-(dichloromethyl)pyrazolo[1,5-*a*][1,3,5]triazine derivatives **79a**–**x**, were tested for anticancer activity against NCI 60 cell lines. The anticancer evaluation results disclosed that the five products **79e**, **79f**, **79i**, **79u**, and **79v** effectively inhibit the growth of non-small cell lung, colon, and renal cancer cell lines [124].

**Scheme 26:**
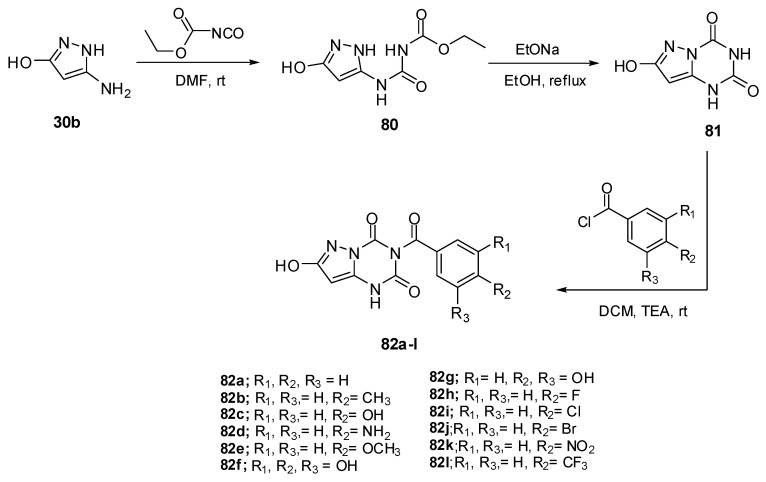
The synthesis route of pyrazolo[1,5-*a*][1,3,5]triazine derivatives **82a**–**l**.

Raghu et al. (2023) synthesized and produced a series of pyrazolo[1,5-a][1,3,5]triazine derivatives **82a**–**l**. They started by reacting 5-aminopyrazole derivative **30b** with ethoxycarbonyl isocyanate to obtain ethyl pyrazole carbamate derivative **80**. Further treatment of derivative **80** with sodium ethoxide resulted in the formation of pyrazolo[1,5-*a*][1,3,5]triazine-2,4-dione derivative **81** (82% yield). Finally, compound **81** was reacted with substituted benzoyl chloride derivatives yielding pyrazolo[1,5-*a*][1,3,5]triazine derivatives **82a**–**l** (85–93% yields) (Scheme 26). All the products, pyrazolo[1,5-*a*][1,3,5]triazine derivatives **82a**–**l**, were assessed for antioxidant activity (DPPH radical scavenging). The antioxidant evaluation results revealed that the four products **82c** (IC_50_ = 88.21 ± 0.55 µM), **82d** (IC_50_ = 99.04 ± 0.63 µM), **82f** (IC_50_ = 80.33 ± 0.49 µM), and **82g** (IC_50_ = 84.65 ± 0.55 µM) possess the highest levels of antioxidants compared to the standard drug ascorbic acid (IC_50_ = 92.63 ± 0.63 µM) [125].

### 4.6. Pyrazolo[3,4-d][1,2,3]triazine Derivatives

Pyrazolo[3,4-*d*][1,2,3]triazine derivatives possess various pharmacological activities such as antioxidant and antitumor effects [126]. Here, we will present an example of the synthesis method and biological activity of pyrazolo[3,4-*d*][1,2,3]triazine derivatives.

**Scheme 27:**
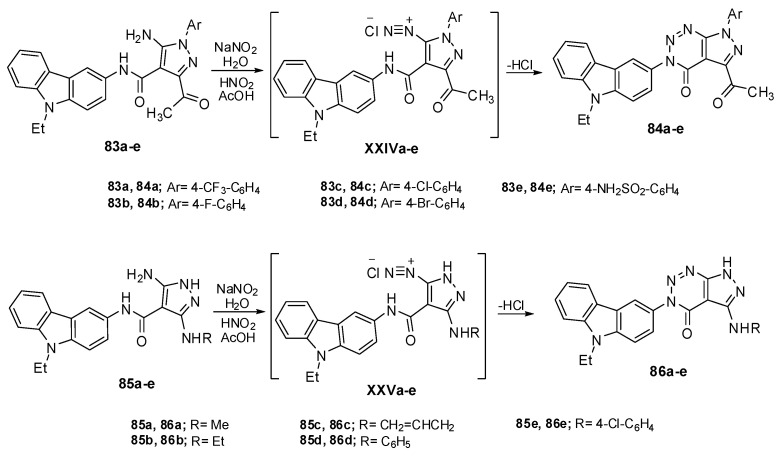
The synthesis route of pyrazolo[3,4-*d*][1,2,4]triazine derivatives **84a**–**e** and **86a**–**e**.

Bondock et al. (2021) reported the synthesis of some new pyrazolo[3,4-*d*][1,2,4]triazine derivatives containing the carbazole moiety. They prepared two series of pyrazolo[3,4-*d*][1,2,3]triazin-4-one derivatives **84a**–**e** (64–69% yields) and **86a**–**e** (82–88% yields) via the diazotization of 3-acetyl-5-aminopyrazole derivatives **83a**–**e** and 5-amino-3-(substituted amino)-pyrazole derivatives **85a**–**e**, respectively (Scheme 27). The two series, pyrazolo[3,4-*d*][1,2,3]triazin-4-one derivatives **84a**–**e** and **86a**–**e**, were assessed for anticancer activity (HCT-116, HepG-2, and MCF-7). The anticancer evaluation results revealed that the three products, **86b**, **86d**, and **86e**, possess weak cytotoxicity against all tested cancer cell lines in comparison with doxorubicin, while the other derivatives displayed no cytotoxicity [127].

### 4.7. Pyrazolo[5,1-c][1,2,4]triazine Derivatives

Pyrazolo[5,1-*c*][1,2,4]triazine derivatives have demonstrated a range of pharmaceutical benefits, including antimicrobial [128,129,130], potent RNA polymerase inhibitors [131], anticancer [132], and antiviral [133] properties. Here, we will discuss some of the synthesis methods and biological activities of pyrazolo[5,1-*c*][1,2,4]triazine derivatives.

**Scheme 28:**
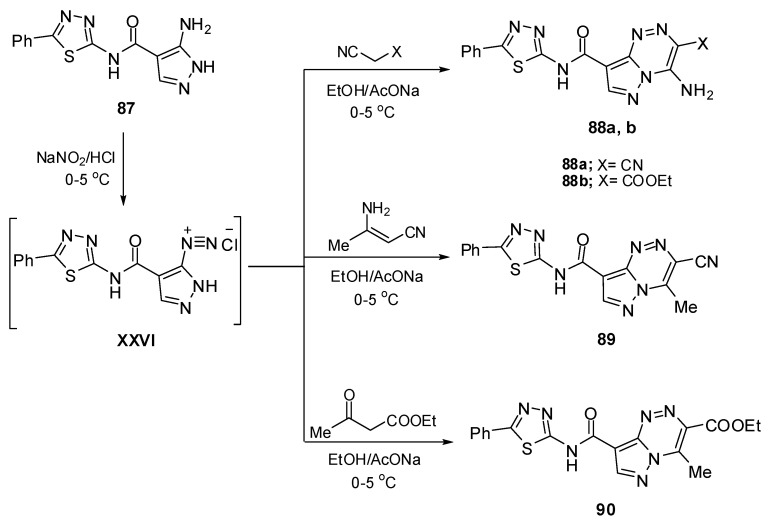
The synthesis route of pyrazolo[5,1-*c*][1,2,4]triazine derivatives **88a**,**b**, **89**, and **90**.

In 2024, Abu-Melha reported the synthesis of new thiadiazole-pyrazolo[5,1-*c*][1,2,4]triazine derivatives **88a** (81% yield), **88b** (78% yield), **89** (62% yield), and **90** (86% yield) through the diazotization of 5-aminopyrazole-thiadiazole derivative **87** and then coupling with malononitrile, ethyl cyanoacetate, 3-aminobut-2-enenitrile, and ethyl acetoacetate, respectively (Scheme 28). The thiadiazole-pyrazolo[5,1-*c*][1,2,4]triazine derivatives were assessed for in vitro anticancer activity (PC3, HCT-116, HepG-2, and MCF-7). The anticancer evaluation results revealed that product **90** (IC_50_ = 7.53 ± 0.08 μM) possesses strong anticancer activity towards the MCF-7 cell line in comparison with 5-fluorouracil (5-FU) (IC_50_ = 5.39 ± 0.52 μM) [134].

**Scheme 29:**
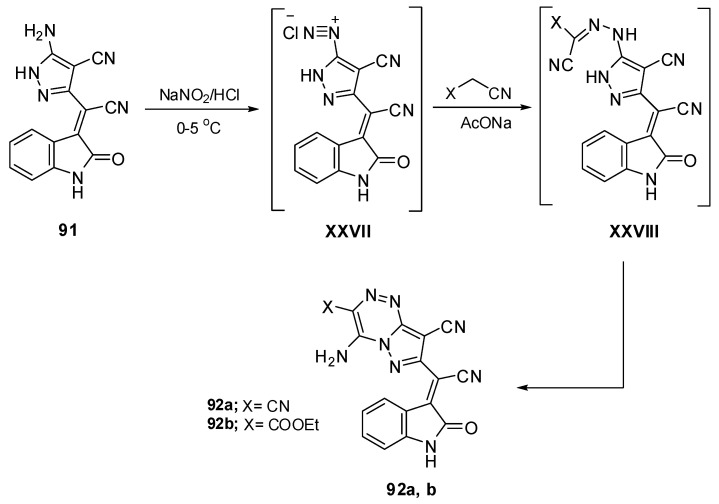
The synthesis route of 2-oxoindolin-3-ylidene-pyrazolo[5,1-*c*][1,2,4]triazines **92a**,**b**.

The novel 2-oxoindolin-3-ylidene-pyrazolo[5,1-*c*][1,2,4]triazine derivatives **92a** (68% yield) and **92b** (70% yield) were prepared through the diazotization of 5-amino-(2-oxoindolin-3-ylidene)methyl-1*H*-pyrazole derivative **91** and then coupling with malononitrile or ethyl cyanoacetate, respectively (Scheme 29). The oxoindolin-3-yl-pyrazolo[5,1-*c*][1,2,4]triazine derivatives **92a**,**b** were assessed for antimicrobial activity. The antimicrobial evaluation results revealed that product **92a** possesses moderate antibacterial activity towards *Staphylococcus aureus* (*S. aureus*), *Pseudomonas aeruginosa* (*P. aeruginosa*), and *Klebsiella pneumoniae* (*K. pneumoniae*) in comparison with the reference drug, chloramphenicol [135].

**Scheme 30:**
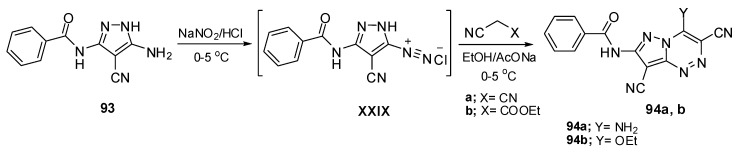
The synthesis route of pyrazolo[5,1-*c*][1,2,4]triazines **94a**,**b**.

In 2020, Hebishy et al. synthesized *N*-(pyrazolo[5,1-*c*][1,2,4]triazin-7-yl)benzamides **94a** (90% yield) and **94b** (75% yield) as antiviral agents against bird flu influenza (H5N1) through the diazotization of *N*-(5-aminopyrazol-3-yl)benzamide derivative **93** and then coupling with malononitrile or ethyl cyanoacetate, respectively (Scheme 30). The antiviral evaluation results revealed that the two products **94a** and **94b** possess antiviral activities against bird flu influenza (subtype H5N1) with a viral reduction of 50% and 46.67%, respectively [136].

## 5. Pyrazole-Based Derivatives and Their Biological Activities

Pyrazole-based derivatives possess diverse valuable biological activities [137,138,139,140] such as anti-cancer and anti-tuberculosis properties [141,142], antimicrobial effects [143], DNA gyrase inhibition [144], and anti-diabetic potential [145]. Here, we will survey some pyrazole-based Schiff bases and their associated biological activities as examples of pyrazole-based derivatives.

**Scheme 31:**
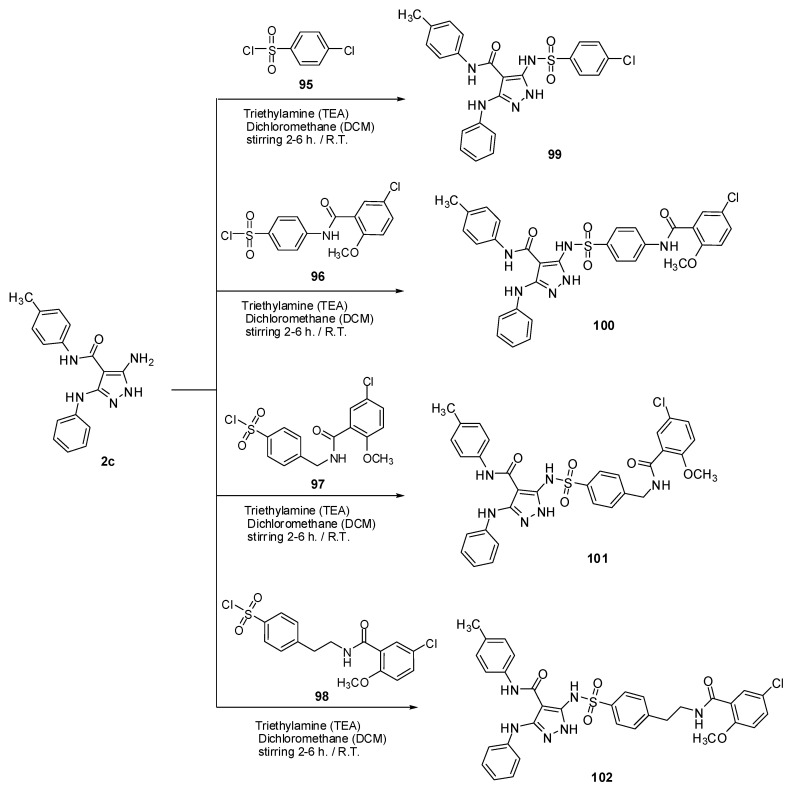
The synthesis route of sulfonamide-pyrazole derivatives **99**–**102**.

Abdelazeem et al. (2024) designed and prepared a new series of sulfonamide derivatives based on 5-aminopyrazole as anti-diabetic and anti-Alzheimer agents. The sulfonamide derivatives **99**–**102** (85–98% yields) were prepared through the reaction of 5-amino pyrazole derivative **2c** with some sulfonyl chloride derivatives **95**–**98** [4-chlorobenzene-1-sulfony lchloride (**95**), 4-(5-chloro-2-methoxybenzamido)benzene-1-sulfonyl chloride (**96**), 4-((5-chloro-2-methoxybenzamido)methyl)benzene-1-sulfonyl chloride (**97**), and 4-(2-(5-chloro-2-methoxybenzamido)ethyl)benzene-1-sulfonyl chloride (**98**)] (Scheme 31). The anti-diabetic and anti-Alzheimer evaluation results revealed that some sulfonamide-pyrazole derivatives possess anti-diabetic and anti-Alzheimer activities [146].

## 6. Conclusions and Future Perspectives

In conclusion, we have discussed a range of synthetic methods and potential biological activities of fused pyrazole and pyrazole-based derivatives that have been developed in the past 5 years, drawing inspiration from 5-aminopyrazole derivatives.

Fused pyrazoles, including pyrazolopyridine, pyrazolopyrimidine, pyrazoloquinazoline, and pyrazolotriazine derivatives, have shown diverse biological activities and medicinal applications.

Fused pyrazoles have demonstrated properties such as antioxidants, anticancer, enzyme inhibitors, antimicrobials, and anti-tuberculosis activities. As a result, fused pyrazoles hold a significant position in the fields of organic, pharmaceutical, and medicinal chemistry.

Fused pyrazole derivatives were prepared through traditional reactions or a facile one-pot multi-component reaction. In the future, nanotechnology is a promising strategy and we will utilize this technique for organic synthesis. Additionally, green chemistry will be applied widely for organic synthesis.

Fused pyrazole derivatives associated with moieties such as isatin, thiadiazole, carbazole, piperazine, or aryl azo may increase their biological potency. Therefore, the molecular hybridization strategy will be utilized for the synthesis of fused pyrazole derivatives with bioactive fragments for the production and discovery of new drugs.

Finally, this study will assist research groups in the fields of organic, bioorganic, pharmaceutical, and medicinal chemistry in the design and development of potent new drugs that are more selective and less toxic.

## Abbreviations

AD	Alzheimer’s disease
ALK	Anaplastic lymphoma kinase
*B. megaterium*	*Bacillus megaterium*
CCD-18Co	Normal colon fibroblast cell line
COX-2	Cyclooxygenase-2 enzyme
CS_2_	Carbon disulfide
DCM	Dichloromethane
DMF	Dimethylformamide
DPP-4 enzyme	Dipeptidyl peptidase-4 enzyme
*E. coli*	*Escherichia coli*
EO771	Breast cancer cell line
EtOH	Ethanol
5-FU	5-Fluorouracil
HCT 116	Colorectal carcinoma cell line
Hela	Cervical cancer cell line
Hep-2	Laryngeal cancer cell line
HT-29	Colorectal adenocarcinoma cell line human-derived
*K. pneumoniae*	*Klebsiella pneumoniae*
KPC	Pancreas cancer cell line
5-LOX	5-Lipoxygenase enzyme
MCF-7	Breast cancer cell line
MDRB	Multidrug resistance in bacteria
MEFs	Non-cancerous murine cell line of mouse embryonic fibroblasts
MSC	Melanoma skin cancer
MWI	Microwave irradiation
NCI	National Cancer Institute
NPs	Nano-particles
NSAID	Non-steroidal anti-inflammatory drug
NSCLC	Non-small cell lung carcinoma
*P. aeruginosa*	*Pseudomonas aeruginosa*
PC-3	Prostatic cancer cell line
PDE5	Phosphodiesterase-5
PGD2	Prostaglandin D2
PGE2	Prostaglandin E2
PGF2	Prostaglandin F2
PGI2	Prostacyclin
ROS1	c-ros oncogene 1
RSV	Respiratory syncytial virus
*S. aureus*	*Staphylococcus aureus*
*S. Typhi*	*Salmonella typhi*
TEA	Triethylamine
TXA2	Thromboxane
VEGFR-2	Vascular Endothelial Growth Factor Receptor
WI38	Normal fibroblast cells
